# Neural Tracking of Sustained Attention, Attention Switching, and Natural Conversation in Audiovisual Environments Using Wearable EEG

**DOI:** 10.1111/ejn.70538

**Published:** 2026-05-08

**Authors:** Johanna Wilroth, Oskar Keding, Martin A. Skoglund, Maria Sandsten, Martin Enqvist, Emina Alickovic

**Affiliations:** ^1^ Department of Electrical Engineering Linköping University Linköping Sweden; ^2^ Centre for Mathematical Sciences Lund University Lund Sweden; ^3^ Eriksholm Research Centre Oticon A/S Snekkersten Denmark

**Keywords:** audiovisual stimuli, auditory attention decoding, conversation, neural speech tracking, wearable EEG

## Abstract

Everyday communication is dynamic and multisensory, often involving shifting attention, overlapping speech, and visual cues. Yet, most neural attention tracking studies are still limited to highly controlled lab settings, using clean, often audio‐only stimuli and requiring sustained attention to a single talker. This work addresses that gap by introducing a novel dataset from 24 normal‐hearing participants. We used a wearable electroencephalography (EEG) system (44 scalp electrodes and 20 cEEGrid electrodes) in an audiovisual (AV) paradigm with three conditions: sustained attention to a single talker in a two‐talker environment, attention switching between two talkers, and unscripted two‐talker conversations with a competing single talker. Analysis included temporal response functions (TRFs) modeling, optimal lag analysis, selective attention classification with decision windows ranging from 1.1 to 35 s, and comparisons of TRFs for attention to AV conversations versus side audio‐only talkers. Key findings show significant differences in the attention‐related P2 peak between attended and ignored speech across conditions for scalp EEG. Interestingly, our results revealed strong cross‐condition generalization, with models trained in one condition maintaining good performance when evaluated on the other two. No significant change in performance between switching and sustained attention suggests robustness for attention switches. Optimal lag analysis revealed a narrower peak for conversation compared to single‐talker AV stimuli, reflecting the additional complexity of multi‐talker processing. Classification of selective attention was consistently above chance (55%–70% accuracy) for scalp EEG, whereas cEEGrid data yielded lower correlations, highlighting the need for further methodological improvements. These results demonstrate that wearable EEG can reliably track selective attention in dynamic, multisensory listening scenarios and provide guidance for designing future AV paradigms and real‐world attention tracking applications.

## Graphical abstract

Neural tracking‐based electroencephalography (EEG) needs to generalize between listening scenarios. Participants perform various listening tasks, including sustained attention and attention‐switching paradigms, using audiovisual material featuring both single speakers and multi‐speaker conversations. Models based on wearable EEG systems demonstrate robust generalization across different listening tasks and speech material.

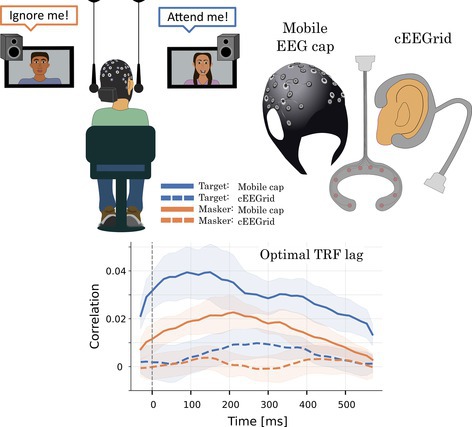

**Abbreviations**
AAD: auditory attention decoding
AV: audiovisual
ConvAC: conversation attention condition
CV: cross‐validation
EEG: electroencephalography
ERP: event‐related potential
GT: gammatone
HL: hearing level
ICA: independent component analysis
SD: standard deviation
SPL: sound pressure level
SustAC: sustained attention condition
SwitAC: switching attention condition
TRF: temporal response function
TV: television

## 1 Introduction

Neurophysiological studies of speech communication have traditionally relied on highly controlled stimuli—short, repetitive sounds or isolated sentences—designed to obtain time‐locked evoked responses such as event‐related potentials (ERPs) (Stapells 2002). Although these controlled paradigms allow for well‐defined experimental contrasts, they fail to capture the neural dynamics involved in real‐world listening. A recent shift has occurred toward using more naturalistic stimuli, largely driven by the development of data‐driven models to decode cognitive states such as auditory attention. This transition reflects a growing ambition to understand and track selective auditory attention in more realistic, everyday listening situations, an essential step toward building EEG‐based auditory attention decoding (AAD) systems that can infer the focus of a listener's attention (O'sullivan et al. 2015; Alickovic et al. 2019; Geirnaert et al. 2021) for future hearing devices (Lunner et al. 2020; Di Liberto and Ip 2025).

Electroencephalography (EEG) has become the primary neuroimaging modality for AAD research due to its portability and compatibility with wearable systems (Debener et al. 2015; Kappel et al. 2018). Yet, despite this potential, current EEG‐based AAD paradigms remain far from reflecting everyday listening. Most studies remain confined to laboratory environments, using clean, often audio‐only stimuli and requiring sustained attention to a single talker for extended periods. Such designs contrast sharply with natural communication, which is multisensory and noisy and often requires switching attention between speakers (Keidser et al. 2020; Bodie 2023). These simplifications limit both the ecological validity and practical relevance for integration into hearing technologies.

The path toward tracking attention in naturalistic settings faces three critical roadblocks. First, there is an over‐reliance on clean, controlled, and often audio‐only speech stimuli (O'sullivan et al. 2015; Schäfer et al. 2018; Ciccarelli et al. 2019; Alickovic et al. 2019; Alickovic et al. 2021; Geirnaert et al. 2021; Straetmans et al. 2024). Most studies use speech recorded by professional speakers, with controlled pauses and minimal background noise, which does not reflect the complexity of real‐world communication. Everyday communication is inherently multisensory (von Kriegstein 2012), often accompanied by visual cues such as lip movements and facial expressions. Recognizing this, recent efforts have started to introduce audiovisual (AV) stimuli into AAD experiments, showing that visual speech cues can enhance neural tracking and decoding of auditory attention (Crosse et al. 2015; O'Sullivan et al. 2019; Fu et al. 2019; Wang et al. 2023; Rotaru et al. 2024). However, these AV paradigms still frequently rely on scripted speech and controlled interactions, which differ from the spontaneous, overlapping, and noisy sound flow of everyday communication. To bridge this gap, AAD paradigms could integrate naturalistic AV speech that captures the dynamic and unscripted nature of real‐life interactions.

Second, there is a limited understanding of how neural speech processing adapts across different listening tasks. Traditional AAD paradigms, which require participants to sustain attention on a single (usually audio‐only) talker for prolonged durations (O'sullivan et al. 2015; Bleichner et al. 2016; Schäfer et al. 2018; Alickovic et al. 2019; Geirnaert et al. 2021; Belo et al. 2021), fail to capture the dynamic shifts in attention characteristic of real‐world listening. Recognizing this gap, recent studies have begun exploring attention‐switching paradigms (Haro et al. 2022; Van de Ryck et al. 2025; Carta et al. 2025). For example, Haro et al. (2022) showed that although attention switches between two audio‐only speakers can be decoded, these switches are associated with increased listening effort. Carta et al. (2025) further examined neural dynamics during attention shifts, observing that neural tracking of a newly attended speaker emerges even before disengagement from the previous one, suggesting a brief period of simultaneous encoding of both streams. Extending these investigations to AV contexts, Van de Ryck et al. (2025) demonstrated that AAD performance during switching trials is comparable to sustained attention trials in multi‐conversation AV settings. However, these studies either focus on neural dynamics or on decoding attention, but not together during attention switches in complex AV conversations. This leaves a gap in understanding how attention decoding performs when moving from simple two‐talker tasks to more natural, multi‐talker conversations.

Third, achieving realistic AAD requires more compact and less intrusive EEG systems. Wearable EEG systems, capable of recording neural activity using either full scalp electrode arrays or more discreet in/around‐ear electrodes (e.g., in‐ear EEG [Kappel et al. 2018] or cEEGrid [Debener et al. 2015] arrays), offer a promising solution for capturing neural responses in ecologically valid environments. Wearable EEG caps and cEEGrid arrays have been used in settings outside the laboratory (Straetmans et al. 2024), where participants walked outdoors while listening to competing speech streams. In that study, significantly stronger neural tracking of the speech envelope was observed for the attended speaker compared to the ignored speaker when using a wearable EEG cap, demonstrating that selective auditory attention can be decoded in naturalistic, active environments.

In contrast, results based on cEEGrid recordings in conversational AV settings have not yet been reported. The cEEGrid, a flexible electrode array positioned around the ear, has nevertheless been shown to capture neural signatures of auditory attention. However, its application has largely been limited to simplified experimental paradigms, most commonly involving sustained attention to a single talker over extended durations (Bleichner et al. 2016; Holtze et al. 2022; Mirkovic et al. 2016). Moreover, current cEEGrid‐based AAD approaches often rely on individual parameter tuning, which may introduce subject‐specific biases in performance evaluation.

These three roadblocks highlight that the literature rarely integrates natural AV conversation with dynamic attentional demands, especially within EEG frameworks suitable for studying speech perception as a communicative and dynamic real‐world process. To address this gap, we integrated naturalistic AV conversation, attention switching, and wearable EEG technology. EEG from 24 normal‐hearing participants using 44 scalp electrodes and cEEGrid arrays was recorded across three conditions: sustained attention to one of two competing talkers, attention switching between two competing talkers, and attending to a front conversational AV source with a side competing single talker. To overcome the first roadblock of limited ecological validity, we took a step toward more realistic experimental settings by using naturalistic AV stimuli. To address the second roadblock of dynamic attention demands, we included both sustained and switching attention tasks, as well as attention to conversation, each with competing, ignored speech present. To tackle the third roadblock of limited portability and practical EEG application, we employed wearable EEG recording with both scalp and around‐the‐ear cEEGrid electrodes. Notably, the study was conducted in Danish, a language not previously examined in this context, enabling assessment of potential language‐specific effects.

Neural attention tracking was analyzed using speech‐to‐EEG correlation‐based methods (Alickovic et al. 2019; Geirnaert et al. 2022; Brodbeck et al. 2023), specifically temporal response functions (TRFs)—linear filters fitted between speech features and simultaneously recorded EEG signals. This allowed us to examine both qualitative TRF characteristics and the ability to decode participants' attention across the three conditions and two EEG modalities. The main novelty of this study lies in the integration of wearable EEG devices within an AV paradigm. A key finding is the observed generalization across conditions: Models trained on one condition performed well when tested on the other two, demonstrating robust cross‐condition transfer. By combining scalp and cEEGrid recordings with naturalistic and dynamic listening tasks, we evaluated how AAD performs in more realistic scenarios, taking steps toward addressing the ecological, dynamic, and practical challenges of everyday listening.

The paper proceeds with a detailed description of the methods in Section 2, covering both data recording and data analysis. Section 3 presents the results, which are then discussed in Section 4. Finally, conclusions and future directions are provided in Section 5.

## 2 Methods and Materials

The experimental protocol was approved by the ethics committee for the capital region of Denmark (Journal Number F‐24047175) and by the Swedish Ethical Review Authority, Sweden (DNR: 2022‐05129‐01). The study was conducted according to the Declaration of Helsinki, and all the participants gave written consent prior to the experiment.

### 2.1 Study Population

The study included 24 native Danish speakers (12 male), aged 23–51 (mean ± SD: 35.7 ± 8.9). All participants had normal hearing, confirmed by otoscopy and audiogram measurements at 250, 500, 1000, 2000, 4000, and 8000 Hz for each ear, with hearing thresholds below 25‐dB HL. Fifteen participants wore glasses or contact lenses.

### 2.2 Paradigm and Experimental Design

**FIGURE 1:**
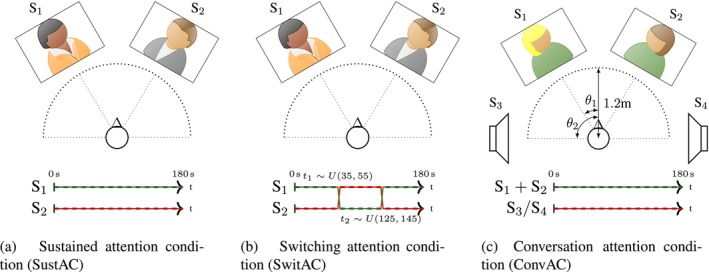
Experiment paradigm and condition designs. Top panels (a)–(c) show the experimental setup of SustAC, SwitAC, and ConvAC, respectively. The setups for SustAC (a) and SwitAC (b) are identical, with attention directed to one out of the two frontal AV speakers. In (c), attention is directed either to the two frontal AV speakers engaged in conversation or to the side single speaker. Below each setup, the instructed attentional focus over the course of a trial is shown: Attention is sustained on a single talker for SustAC and ConvAC, whereas SwitAC includes two switches between speakers.

The experiment took place in a soundproof room under controlled light conditions. Participants sat in a chair in the middle of the room and were presented with AV stimuli from loudspeakers (Genelec 8010A) positioned at ±30°(*θ*_1_) and ±90°(*θ*_2_) azimuth relative to the participants. The two speakers in front (S_1_, S_2_) had computer screens (ThinkVision, 25″), situated directly in front of the speakers. The distance from the screens to the speakers was 1.36 m. Loudspeakers were calibrated at 65‐dB SPL, using single speech with quiet parts longer than 0.8 s cut away. All stimuli were presented with a sample rate of 44.1 kHz. Loudspeakers to each side of the participant (S_3_, S_4_), used in the final experimental condition, had no screens, as the participant was never instructed to look at these speakers. A screen at 0° azimuth, positioned at the same distance as the speaker screens, was used to present instructions to the participant. Chair heights were adjusted so that each participant's vertex aligned naturally with the screens. A schematic of the setup is shown in Figure 1.

#### 2.2.1 AV Stimuli

Two sets of AV stimuli were used in the study: the single talker set and the conversation AV set. The single talker set consisted of excerpts from a Danish TV program with various known personalities sitting and answering questions posed to them from viewers through a live chat. Throughout the trials, speakers primarily looked directly into the camera, with only brief deviations. Four speakers (two male and two female) made up the single talker AV set, discussing various topics related to their professional background.

The conversation AV set consisted of a podcast with two female speakers engaged in conversation, looking at each other. The cameras were positioned to the side of each speaker, so that they faced one another in the frame. Discussion topics included names and friends. For the conversation set, audio from the non‐target talker was attenuated in the opposite talker's loudspeaker to achieve realistic spatialization. When both talkers spoke simultaneously, audio was presented through both speakers. Detailed information about both AV sets is provided in the supporting information.

#### 2.2.2 Procedure and Conditions

Participants completed three tasks across three blocks corresponding to the conditions: sustained attention condition (SustAC), switching attention condition (SwitAC), and conversation attention condition (ConvAC) (see Figure 1). Each trial lasted 180 s.

##### 2.2.2.1 Trial Protocol

A training trial was completed for each condition to ensure participants understood the task. Each trial started with a task instruction, followed by stimuli. At the end of each trial, participants answered three two‐choice questions related to the content of the attended stimuli and rated the listening and understanding difficulty on a 1–7 scale. Self‐determined breaks were allowed after every second trial. Task instructions were presented visually (arrow on the center screen) and with a green dot below the attended speaker's face.

##### 2.2.2.2 SustAC

Eight trials consisted of two competing single talkers from the single talker set (S_1_ and S_2_) presented audiovisually (Figure 1a). Participants were instructed to maintain attention on one speaker for the entire trial while ignoring the other. A green attention target circle remained below the face of the attended speaker throughout the trial. Participants were asked to follow the attended speaker naturally with eye movement, as they would in real life.

##### 2.2.2.3 SwitAC

Also consisting of eight trials, the setup was similar to SustAC, but participants switched attention between the two speakers (S_1_ and S_2_) twice per trial (Figure 1b). Switch times were randomly drawn from the uniform distribution *U*(35,55) s and *U*(125,145) s, respectively. Switches were cued by changing the green attention target circle to yellow 2 s before the switch; at switch onset, the circle moved to the other screen and reverted to green. Center‐screen instructions were updated to indicate the newly attended speaker. Participants were instructed to naturally follow the currently attended speaker with their eyes and to shift gaze to the newly attended speaker after each switch.

##### 2.2.2.4 ConvAC

The setup differed, presenting a two‐speaker conversation from the conversation set at S_1_ and S_2_, alongside a competing single speaker from the single talker set (S_3_ or S_4_), evenly randomized across trials (Figure 1c). Participants were instructed to attend either the conversation or the side single talker. When attending the single talker, participants maintained their gaze fixation on a point on the center screen, whereas for conversation trials, they could naturally follow the speakers with eye movements while minimizing unnecessary head movements. ConvAC consisted of nine trials, with the extra trial always being attended to the conversation. This was to increase the amount of data available to perform intra‐conversation attention analysis.

For all conditions, competing speakers were always of opposite sex. Stimuli were randomized across participants to avoid biases. Within each condition, the distribution of attention toward male and female speakers, as well as left and right speaker positions, was balanced. After completing all three blocks, participants completed a questionnaire to report any familiarity with the speakers and rate the overall interestingness of each speaker. For SwitAC, the post‐trial comprehension questions were based on speech segments from 0 to 30 s, 60 to 120 s, and 150 to 180 s to avoid overlap with attention switches and cover material from both speakers.

### 2.3 Data Acquisition

#### 2.3.1 Neural Data

**FIGURE 2:**
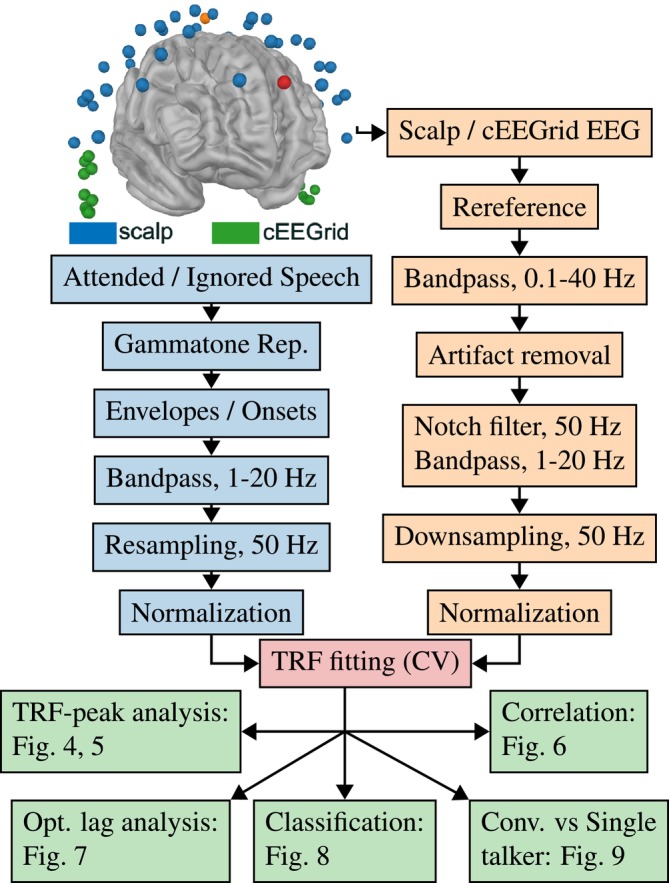
The top‐left plot shows a three‐dimensional rendering of electrode locations projected onto a standard brain model using MNE‐Python. The plot illustrates the EEG setup, including the common mode sense (CMS) and driven right leg (DRL) electrodes in orange and red, respectively. Scalp EEG electrodes are shown in blue, and cEEGrid electrodes are shown in green. The cEEGrid consists of 10 electrodes placed around each ear. In the rendering, the positions of cEEGrid arrays may not be exact. The subsequent preprocessing steps for the stimuli (light blue) and EEG data (light orange) are presented as flowcharts. Forward and backward temporal response function (TRF) models are then fitted using cross‐validation (CV) (red). The forward model is analyzed both qualitatively and quantitatively by inspecting TRF waveforms, whereas the backward model is analyzed quantitatively via correlation‐based metrics, including reconstruction accuracy and classification performance (green).

Neural responses were recorded through a 64‐channel EEG, with a modified Easycap and a Smarting Pro X amplifier. To include cEEGrid, 20 cap electrodes were replaced with a pair of 10‐channel around‐the‐ear arrays. This resulted in 44 scalp EEG electrodes (blue in Figure 2) and 20 cEEGrid electrodes (L1–10 and R1–10, green in Figure 2).

EEG and other measurements were digitally recorded through the associated mbtStreamer application. The sampling rate of the EEG was 500 Hz, with FCZ as the reference electrode. Impedances were visually monitored to remain below 20 kΩ.

#### 2.3.2 Other Data Streams

Two microphones (Behringer ECM‐8000) were placed 10 cm behind each of the participants' ears to capture the sound field (not analyzed in this study). Sound was preamplified (Golden Age Project PRE‐73 Jr MKII) before digital conversion using Ferrofish PULSE16 MX and was subsequently downsampled to 11,025 Hz. Audio was simultaneously captured and sent to the mbtStreamer application. A Tobii Pro Nano eye‐tracking device (Tobii Pro Nano 2025) was positioned in front of the participant to record ocular data, including gaze position, eye movements, and pupil responses. An initial analysis of the eye‐tracking recordings indicated that participants' gaze behavior was consistent with the task instructions across conditions. The focus of the present work is neural speech tracking, and a detailed analysis of the eye‐tracking data falls outside its scope. A pilot study conducted on a single participant prior to completion of data collection showed the feasibility of estimating attended‐speech labels for AAD using eye‐tracking data alone (Wilroth et al. 2025).

#### 2.3.3 Data‐Stream Synchronization

The experiment was run on Windows using the Psychopy package for Python (Peirce et al. 2019). Sound I/O was handled through the Ferrofish PULSE16 MX. Stimuli and data synchronization were performed on a separate computer using LabStreamingLayer (*sccn/labstreaminglayer* 2025), which aligned triggers with EEG and other measured time series across devices over the local network.

### 2.4 Data Preprocessing

Neural data were initially separated into scalp EEG and cEEGrid EEG. Scalp EEG was rereferenced to the average of all electrodes, and cEEGrid EEG was rereferenced to the L4 and R4 electrodes (Mirkovic et al. 2016). Although scalp EEG and cEEGrid data possess different signal‐to‐noise ratios and spatial characteristics, we adopted a uniform preprocessing pipeline to maintain methodological consistency. Preliminary evaluations of alternative strategies, including rereferencing to the average of all cEEGrid electrodes instead of L4 and R4, processing both with and without ICA, and varying bandpass filter settings (e.g., 1–10 Hz), did not result in significant performance gains. We therefore processed both data streams in parallel (see Figure 2) to enable a direct comparison of the recording modalities, while leaving modality‐specific optimization for future study. Preprocessing included notch‐filtering at 50 Hz to remove line noise, followed by bandpass filtering between 0.1 and 40 Hz to remove EEG drifts and unwanted noise. Independent component analysis (ICA) using the extended infomax algorithm (Lee et al. 1999) was then applied with 20 components for scalp EEG and 10 components for cEEGrid EEG. Manual inspection and removal of artifactual components resulted in an average of five components removed for scalp EEG and three for cEEGrid EEG. Following the methodology described in Bleichner and Debener (2019), ICA components corresponding to artifacts in cEEGrid data were identified conservatively. Eye‐movement and cardiac components were removed based on their characteristic time‐domain patterns, whereas muscle artifacts were identified by elevated spectral power above 20–30 Hz. Due to the limited spatial separation and higher noise level of cEEGrid electrodes, components were only removed when the artifact signature was clearly identifiable. Furthermore, to assess the unmixing quality of ICA for cEEGrid data, the explained variance of the 10 cEEGrid components was 89%, and the average correlation between unmixing matrices across five ICA reruns was 0.99. A decrease in mutual information after unmixing was also observed, indicating stable component separation without evidence of substantial reconstruction bias. After component reprojection, EEG data were bandpass‐filtered between 1 and 20 Hz, downsampled to 50 Hz, and normalized (see Figure 2). Following recommendations for filter reporting in electrophysiology (Widmann et al. 2015), our preprocessing was implemented using the Eelbrain pipeline (Brodbeck et al. 2023), which wraps MNE‐Python's finite impulse response (FIR) filtering routines (Gramfort et al. 2013). Linear‐phase FIR bandpass filters were designed using a Hamming window (firwin) and applied in a zero‐phase (non‐causal) manner by applying the filter forward and backward in time. The lower and upper cutoff frequencies were specified for each processing step (0.1–40 Hz and 1–20 Hz). Filter length and transition bandwidth were automatically determined based on the data sampling rate (50 Hz) and MNE‐Python's default settings.

Speech feature preprocessing began with gammatone (GT) filtering of the presented audio. At each time point, the GT representations included 128 frequency bands spaced according to the equivalent rectangular bandwidth scale between 80 and 15,000 Hz, with 1‐ms temporal resolution, implemented in Eelbrain. Two representations were derived: the acoustic envelope, computed as the sum of the absolute values of the GT representation across frequency bands, and acoustic onsets, extracted using the acoustic edge detection method (Fishbach et al. 2001; Brodbeck et al. 2020), implemented in Brodbeck et al. (2023). This approach was compared to an alternative method based on the half‐wave rectified derivative with a Savitzky–Golay filter, which yielded similar results, and the half‐wave rectified method was therefore omitted. Both speech representations were subsequently bandpass‐filtered between 1 and 20 Hz, downsampled to 50 Hz, and normalized.

### 2.5 Data Analysis

Neural tracking of speech was modeled using the TRF, a linear FIR model (O'sullivan et al. 2015; Alickovic et al. 2019). Both forward and backward TRF models were used to relate time‐varying speech features to concurrent EEG signals. Let the TRF for channel i be $h\left(i\right)={\left[h\left(l_{1},i\right),h\left(l+1,i\right),\ldots ,h\left(l_{2},i\right)\right]}^{T}$ over time‐lags *l*_1_ to *l*_2_ chosen between cutoff time‐lags [*t*_*min*_,*t*_*max*_]. The forward TRF model predicts the EEG signal ${\hat{y}}_{i}\left(k\right)$ for channel *i* from the speech feature $x\left(k\right)$:

(1) $$\hat{y_{i}}\left(k\right)=\sum_{l=l_{1}}^{l_{2}}h\left(l,i\right)x\left(k-l\right).$$

The backward TRF model reconstructs the speech feature $\hat{x}\left(k\right)$ from the EEG signal *y*_*i*_(*k*):

(2) $$\begin{array}{c} \hat{x}\left(k\right)=\sum_{i=1}^{n_{\mathrm{ch}}}\sum_{l=l_{1}}^{l_{2}}h\left(l,i\right)y_{i}\left(k+l\right). \end{array}$$

TRF estimation in Equations (1) and (2) was performed using the boosting algorithm (Zhang and Yu 2005; David et al. 2007) in Eelbrain (Brodbeck et al. 2023), which promotes sparse, interpretable solutions. Robustness was further enhanced by expressing the TRF as a weighted sum of basis functions, $h\left(l,i\right)=\sum_{p=1}^{P}w_{p}\;\phi_{p}\left(l\right)$, where basis weights *w*_*p*_ are estimated instead of raw TRF coefficients $h\left(l,i\right)$. The basis functions *ϕ*_*p*_ consist of Hamming windows with 50‐ms width, centered around each element of the TRF. Because the sampling rate was 50 Hz, consecutive samples (and thus basis centers) were spaced 20 ms apart. Because each window spans 50 ms, the resulting estimate is smoothed over roughly this time range, which is sufficient to capture the temporal dynamics of the P1, N1, and P2 components. Filters were estimated over time lags ranging from −1 to 1 s, capturing the full lag range in both the forward and backward directions, and model parameters were obtained by minimizing the mean absolute error for each regression problem.

#### 2.5.1 Correlation Metrics and Optimal Lag Analysis

Correlations between model estimates and measured signals are consistently stronger for attended speech than for ignored speech, providing a robust marker of selective attention (O'sullivan et al. 2015). In the forward model (Equation 1), predictions of EEG activity were correlated with recorded EEG, whereas in the backward model (Equation 2), reconstructions of the speech envelope were correlated with the actual speech stimuli. Correlations were computed using the Pearson correlation coefficient:

(3) $$\begin{array}{c} \rho_{x,y}=\frac{\sum_{k}\left(x\left(k\right)-\bar{x}\right)\left(y\left(k\right)-\bar{y}\right)}{\sqrt{\sum_{k}{\left(x\left(k\right)-\bar{x}\right)}^{2}\sum_{k}{\left(y\left(k\right)-\bar{y}\right)}^{2}}} \end{array}$$

Following the observation of no significant difference between attended and ignored speech in the backward correlations of ConvAC (scalp electrodes with acoustic envelope, Figure 6C), we performed further analyses to determine if the pattern remained consistent across all time lags. Specifically, an optimal time‐lag analysis identified the lag intervals providing the highest reconstruction and prediction correlations. Replicating the method in Mirkovic et al. (2016), forward and backward filters were fitted for multiple sliding time‐lag windows of length 45 ms, with 30 ms overlap, in a range from −600 to 600 ms. Correlation and performance metrics were averaged across cross‐validation (CV) folds and EEG electrodes (for the forward model), resulting in one estimate per subject for each sliding window.

#### 2.5.2 Statistical Testing and CV

Tracking auditory attention can further be improved by analyzing components of the TRF waveforms. TRFs estimated from attended speech exhibit distinct components compared to those estimated from ignored speech, reflecting underlying cognitive processes engaged during attention. These components can be statistically compared to identify significant differences between predictions from attended versus ignored speech. Mass‐univariate statistics were employed using independent samples *t*‐tests (Brodbeck et al. 2023), with threshold‐free cluster enhancement correcting for multiple comparisons (Smith and Nichols 2009). TRFs were smoothed with Gaussian kernels of width 50 ms to improve sensitivity. To test correlation metrics, paired sample *t*‐tests were used, with multiple comparisons corrections employed to control for the family‐wise error rate.

All CV‐based performance metrics were obtained using a leave‐one‐trial‐out strategy to prevent overfitting. This approach avoids bias arising from temporal similarity within the same trial, which has been shown to affect AAD performance (Tanveer et al. 2024; Puffay et al. 2023).

## 3 Results

### 3.1 Behavioral Data Analysis

**FIGURE 3:**
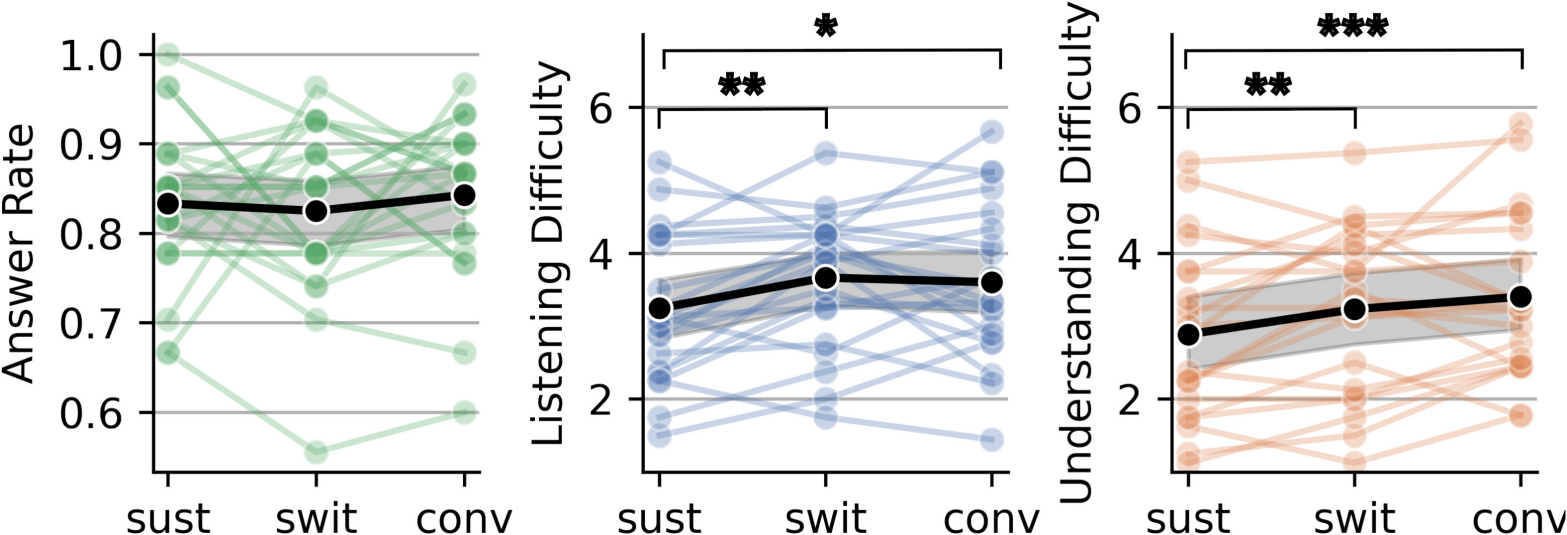
Behavioral analysis: answer rate (left), self‐rated listening difficulty (middle), and self‐rated understanding difficulty (right) for the three conditions, SustAC, SwitAC, and ConvAC. Colored dots show individual subject averages; black markers indicate the group mean. Ratings are on a 1–7 scale. Asterisks denote significance levels (*p < 0.1, **p < 0.05, ***p < 0.01). Significance was corrected for multiple comparisons using the Benjamini–Hochberg procedure (Benjamini and Hochberg 1995).

To confirm that participants performed the listening tasks as instructed across the three conditions, the percentage of correctly answered questions was calculated (Figure 3, left). Response accuracy was 85% for SustAC, 84% for SwitAC, and 89% for ConvAC. This indicates that participants focused on the attended talker and understood the speech material.

Self‐rated difficulty was recorded on a 1–7 scale after each trial, separately for listening difficulty (Figure 3, middle) and understanding difficulty (Figure 3, right), with 1 representing the lowest difficulty. Individual averages are shown in color, and overall averages are shown in black. Paired *t*‐tests revealed a significant increase in listening difficulty from SustAC to SwitAC and ConvAC (*p <* 0.05, *p <* 0.1). Similarly, both SwitAC and ConvAC were rated as significantly more difficult to understand than SustAC (*p <* 0.05, *p <* 0.01).

### 3.2 Neural Data Analysis

**FIGURE 4:**
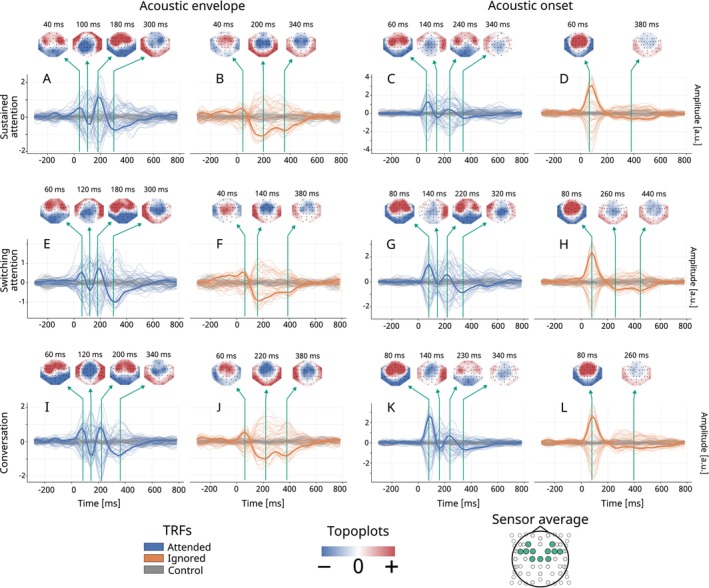
TRF peak analysis. TRFs for our three conditions SustAC (A–D), SwitAC (E–H), and ConvAC (I–L), shown for attended (blue) and ignored (orange), with random control speech (gray). The left two and right two columns show TRFs for the acoustic envelope and the acoustic onset, respectively. The topographic plots show spatial patterns at observed peaks of interest, where red indicates positive amplitude and blue negative amplitude. All presented plots are for the scalp EEG sensors, with the thicker lines showing the TRF channel‐average, as indicated by the cap layout at the bottom.

**FIGURE 5:**
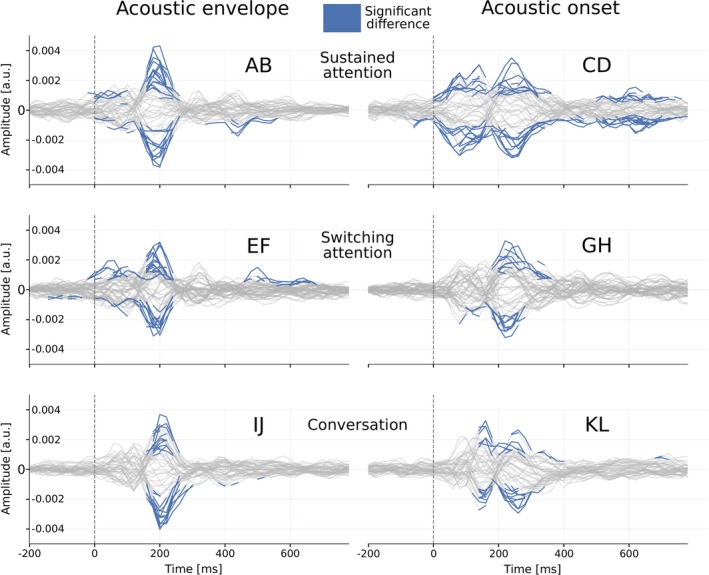
TRF cluster analysis: statistical cluster analysis on the differences between attended and ignored TRFs presented in Figure 4. AB is the result from Figure 4 A,B,CD from Figure 4 C,D, and so forth. The analysis used an independent samples *t*‐test in Eelbrain, where *p <* 0.05 clusters are shown in blue.

Neural responses to attended and ignored speech were analyzed using forward and backward models and classification metrics. Figure 4 shows average forward TRFs across the three conditions (SustAC, SwitAC, and ConvAC) for attended (blue), ignored (orange), and control (gray) speech, with separate columns for the acoustic envelope and acoustic onset features. Individual electrodes are shown as thin lines, with the average over 11 electrodes (F3, F4, FC1–FC6, C1, Cz, and C2) bolded. These electrodes were selected as they cover fronto‐central regions, which are commonly implicated in AAD and speech‐tracking studies (Di Liberto et al. 2015; Crosse et al. 2016). Topoplots illustrate scalp distributions at peaks of interest. Figure 5 presents statistical cluster analysis, highlighting significant differences between attended and ignored TRFs.

**FIGURE 6:**
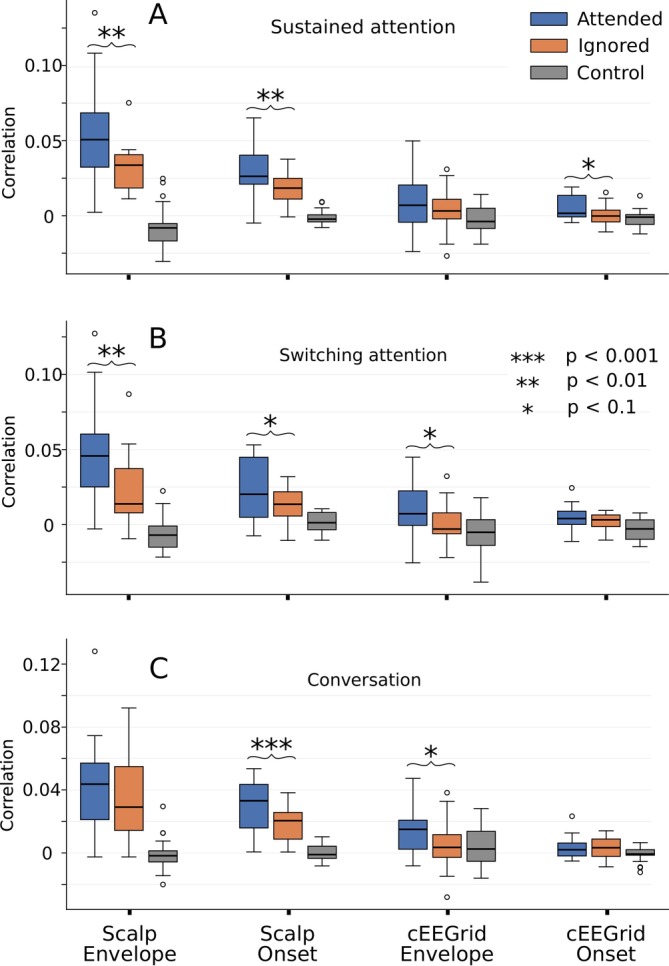
Correlation analysis: Pearson's correlation between reconstructed and real speech (backward model, 35 s) for SustAC (A), SwitAC (B), and ConvAC (C). The *x*‐ticks indicate electrode type (scalp, cEEGrid) and speech features (acoustic envelope, acoustic onset). Boxplots show attended (blue), ignored (orange), and control (gray) speech, with the median as the black line. Significance stars mark differences of means between attended and ignored speech. No statistical tests were performed between conditions and control speech.

**FIGURE 7:**
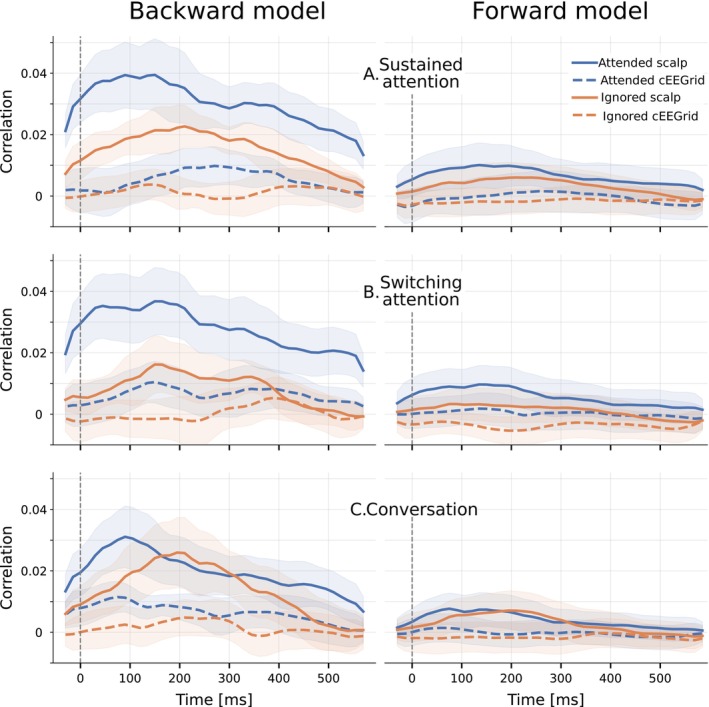
Optimal lag analysis: Pearson's correlation from backward (left) and forward (right) models for SustAC (A), SwitAC (B), and ConvAC (C), using a 45‐ms overlapping time‐lag windows. Each plot shows the attended (blue) and ignored (orange) speech envelope, with scalp electrodes (solid lines) and cEEGrid electrodes (dashed lines). The 95% confidence interval is shaded for each condition. Forward model correlations are averaged across all 44 scalp and 20 cEEGrid electrodes.

**FIGURE 8:**
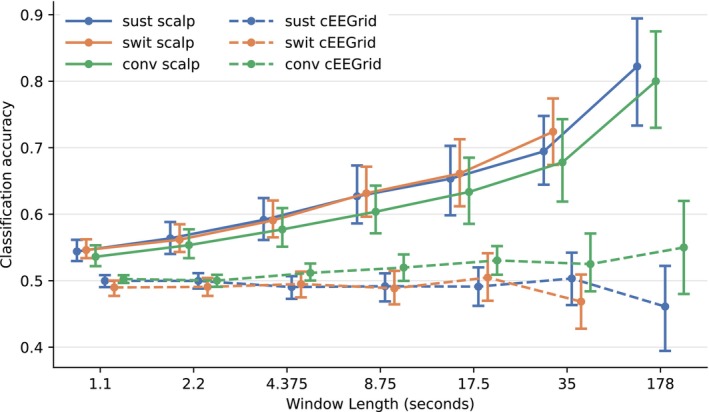
Attention classification analysis: average Pearson's correlations and confidence intervals across participants, for backward models applied on data of different lengths. For SwitAC, no values are shown for the 178‐s window, as attention is not sustained for that duration.

**FIGURE 9:**
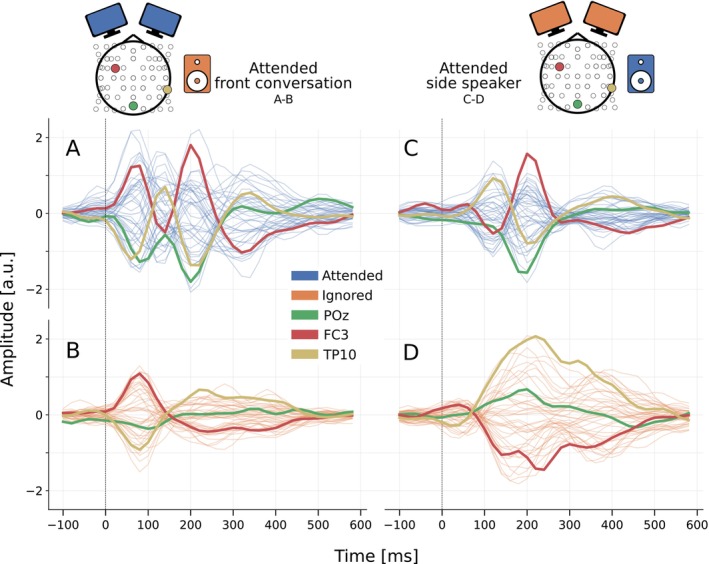
Attention to conversation versus side talker: conversation attention analysis for trials directed to the front conversation (A–B) and to the side speaker (C–D). Attended speech is shown in blue (A,C) and ignored speech in orange (B,D). Three sensors of interest are highlighted: POZ, FC3, and TP10. All TRFs use the acoustic envelope using scalp EEG.

Reconstruction correlations from backward models (based on 35‐s segments) are presented in Figure 6, and optimal lag analysis for forward and backward models is shown in Figure 7. Attended versus ignored speech classification results are presented in Figure 8. Lastly, trials in ConvAC were separated between attention to a front conversation and attention to a side talker, with the resulting TRFs compared in Figure 9.

The results from the three conditions are discussed below.

#### 3.2.1 Sustained Attention (SustAC)

Top row of Figure 4 shows the TRFs for SustAC. For the acoustic envelope (Figure 4A,B), attended speech (Figure 4A) shows peaks for P1 ∼ 40 ms, N1 ∼ 100 ms, and P2 ∼ 180 ms, with P2 being the largest. Ignored speech (Figure 4B) shows smaller amplitudes at these latencies, and the cluster reveals significant differences at P2 and around 400 ms (Figure 5AB, *p <* 0.05), consistent with the N400 associated with higher order linguistic processing and working memory capacity (Salisbury 2004; Kutas and Federmeier 2011; Gillis et al. 2023). For the acoustic onset (Figure 4C,D), attended speech (Figure 4C) shows similar peaks but shifted later: P1 ∼ 60 ms, N1 ∼ 140 ms, and P2 ∼ 240 ms, with P1 being the largest. Ignored speech (Figure 4D) shows smaller peak amplitudes, and cluster analysis identifies significant differences in peaks around 100, 240, and 600 ms (Figure 5CD). The differences between the two speech features are discussed in Section 4.2.3.

Reconstruction accuracy (i.e., correlations between reconstructed and actual speech) from the backward model (Figure 6A) showed significantly higher correlations for attended speech (blue) than for ignored speech (orange) for scalp electrodes (*p <* 0.01) with both speech features, whereas for cEEGrid electrodes, significance appeared only for acoustic onsets (*p <* 0.1). Control speech (gray) was not tested.

Optimal lag analysis (Figure 7) was conducted to investigate temporal response characteristics, evaluating the reconstruction/prediction accuracy of short 45‐ms TRF models across multiple time lags (Mirkovic et al. 2016). As shown in Figure 7A for backward (left) and forward (right) models, attended (blue) and ignored (orange) speech, and scalp (solid lines) and cEEGrid (dashed lines) electrodes, the highest correlation occurred for attended speech with scalp electrodes at lag range *τ* ≈ 40–200 ms for both models, corresponding to P1, N1, and P2 peaks (Figure 4A). A small, later rise around 350 ms reflected the N400 component (Figure 5AB). The ignored speech with scalp electrodes (solid orange line) peaked marginally later (220 ms). For cEEGrid electrodes, the separation between attended and ignored speech started after 100 ms and peaked around 250 ms, differing from Mirkovic et al. (2016) and from our results for SwitAC and ConvAC.

Attention classification analysis (Figure 8) determined the successful decoding of attended versus ignored speech. Using the full trial duration (∼3 min) resulted in the highest accuracy (> 80%) for scalp electrodes (solid lines). Performance for scalp sensors was robust, remaining above chance level even with 1.1‐s windows. The low cEEGrid performance (dashed lines) is discussed further in Section 4.2.4.

#### 3.2.2 Switching Attention (SwitAC)

The middle row of Figure 4 shows the TRF‐peak analysis for SwitAC with acoustic envelope (Figure 4E,F) and acoustic onset (Figure 4G,H). Individual electrodes are thin lines, with the average over 11 central‐frontal electrodes bolded. The TRFs generally resemble those of SustAC (Figure 4A–D). However, a consistent 20‐ms latency shift was observed, delaying both the P1 and N1 peaks for the acoustic envelope feature and also delaying the P1 peak for the acoustic onset feature. This latency shift is discussed further in Section 4. Cluster analysis (Figure 5EF) reveals significant differences between attended (Figure 4E,G) and ignored (Figure 4F,H) speech, with the largest cluster around P2 (∼190 ms) and additional clusters covering P1 and N1. A higher order latency cluster with opposite polarity appears between *τ* ∼ 450–700 ms. For acoustic onset (Figure 5GH), the P2‐peak latency is larger than for the acoustic envelope, consistent with the corresponding TRFs in Figure 4.

Reconstruction accuracy (Figure 6B) confirmed the attentional effect for SwitAC. As in SustAC (Figure 6A), the attended speech (blue) had higher correlation than the ignored (orange) speech and the control (gray) speech across all electrode types and speech features. Significant differences between attended and ignored speech were found for scalp electrodes with acoustic envelope (*p <* 0.01) and acoustic onset (*p <* 0.1), as well as for the cEEGrid electrodes with acoustic envelope (*p <* 0.1).

The optimal lag analysis (Figure 7B) for scalp EEG with attended speech showed patterns similar to SustAC. However, ignored speech and attended speech with cEEGrid, most clearly visible for the backward model, showed two distinct peaks (∼150 and ∼350 ms), in contrast to the single dominant peak of 200–300 ms observed in SustAC.

Attention classification analysis for the SwitAC (Figure 8, orange) showed accuracy similar to SustAC (blue). Accuracy for scalp electrodes (solid line) decreased from approximately 72% at a 35‐s window to 55% at a 1.1‐s window. Note that segment lengths were restricted and do not extend to 178 s, as attention was not sustained toward one talker for such a duration. The lower performance for cEEGrid electrodes (dashed line) will be further discussed in Section 4.2.4.

#### 3.2.3 Conversation Attention (ConvAC)

The bottom row of Figure 4 shows the TRF‐peak analysis for ConvAC. Individual electrodes are thin lines, with the average over 11 central‐frontal electrodes bolded. For the attended speech with acoustic envelope (Figure 4I), four peaks showed similar amplitudes, unlike the P2‐dominant pattern in SustAC (Figure 4A). For the acoustic onset (Figure 4K), a strong P1 dominated. Peak latencies generally resembled SwitAC, with P1 and N1 delayed by ∼20 ms relative to SustAC. Cluster analysis confirmed significant attended‐ignored differences: a large cluster centered around P2 (∼200 ms) for the envelope (Figure 5IJ) and clusters at ∼160 and ∼250 ms for the onset (Figure 5KL).

Figure 9 compares TRFs for trials separating attention to the front conversation (left) versus the side talker (right). The top and bottom rows show TRFs for attended and ignored speech, respectively, all for the acoustic envelope speech feature and the scalp EEG sensors. Three interesting sensors are highlighted for deeper analysis in Section 4: POZ, FC3, and TP10. There is a clear similarity of the TRFs between A and D, as well as between B and C. This indicates that the TRFs trained on conversation features share commonalities compared to single speech, regardless of attentional state, which is further discussed in Section 4.2.5.

Figure 6C shows correlations between reconstructed and real speech (i.e., reconstruction accuracy) for ConvAC, comparing attended (blue), ignored (orange), and control (gray) speech. Median correlations are indicated by black lines, shown for scalp and cEEGrid electrodes and two speech features (acoustic envelope and acoustic onset). Attended speech had significantly higher mean correlations than ignored speech for scalp EEG with acoustic onset (*p <* 0.001) and for cEEGrid with acoustic envelope (*p <* 0.1). Interestingly, for scalp EEG with acoustic envelope, the median correlation for attended speech was similar to SwitAC (≈0.043), but the ignored speech median was higher, although showing no significant difference in mean.

Figure 7C presents the optimal lag analysis. For scalp sensors in the backward model, attended speech (blue, solid) peaked around 100 ms, whereas ignored speech (orange, solid) peaked later around 200 ms in both backward and forward models, consistent with the attention‐related P2 response. Notably, the ignored correlations exceeded attended correlations between 180 and 300 ms. For cEEGrid electrodes, correlation of attended speech (dashed blue line) was consistently above ignored speech for the backward model, with peaks at 90, 180, and 350 ms. The largest peak for cEEGrid (correlation ≈ 0.01) occurs earlier for SustAC ∼280 ms, compared to SwitAC ∼150 ms, which also occurs earlier than ConvAC ∼90 ms. A small separation between attended (dashed blue line) and ignored (dashed orange line) speech is visible in the forward model, though correlations remain low.

The classification analysis for the ConvAC in Figure 8 is shown in green, with a solid line (scalp) and a dashed line (cEEGrid). In general, with scalp sensors, the classification accuracy was 1–3 percentage points lower for ConvAC compared to SustAC (blue). The cEEGrid sensor classification, on the other hand, reached above 50% accuracy for window lengths larger than 8 s, although this is not necessarily significant.

#### 3.2.4 Generalization of Models

To assess the generalization of models trained on one condition, backward models fitted on SustAC were applied to SwitAC and ConvAC data. Using a classification approach based on correlation between reconstructed and actual speech over 35 s, mean accuracy was 0.70 (10th percentile *P*_10_ = 0.47, 90th percentile *P*_90_ = 0.89) for SwitAC and 0.66 (*P*_10_ = 0.50, *P*_90_ = 0.78) for ConvAC. These results indicate that models trained on sustained attention generalize reasonably well to other attention conditions.

## 4 Discussion

### 4.1 Key Findings and Contributions

Our main finding is that selective attention can be reliably tracked across diverse listening conditions—from a simple sustained task to a dynamic, conversational setting—using a wearable EEG system with TRF‐based methods. Unlike much of the existing literature, which relies on controlled audio‐only stimuli, our approach incorporates real, uncontrolled AV speech, bringing neural tracking closer to everyday listening. Statistically significant differences in the P2 peak of the TRFs between attended and ignored speech (Figure 5), widely associated with selective attention and often reduced for ignored speech (Luck and Kappenman 2012), confirm that our wearable EEG setup effectively captures attention‐related neural signatures in these complex conditions. We also observed significant clusters around the N400 peak, likely reflecting higher order processing (Kutas and Hillyard 1983; Proverbio et al. 2022).

Notably, our finding that models trained on SustAC and evaluated on SwitAC and ConvAC maintained strong performance demonstrates robust cross‐condition generalization. In addition, our models also proved stable and robust to attention switches and dynamic conversations, demonstrated by clear differences in reconstruction accuracy between attended and ignored speech (Figure 6). This demonstrates the generalizability of our models across conditions, a capability previously unproven for wearable EEG in AV contexts.

Although wearable EEG has been validated for single‐talker speech in various tasks (Zink et al. 2016; Straetmans et al. 2024; Holle et al. 2021), its performance in switching tasks and conversational data, specifically with AV stimuli, was a significant gap, which this work addresses. We also showed that attention can be decoded in all three conditions using wearable EEG, with classification rates of 65%–75% for 35‐s segments and significant rates for segments lasting a few seconds. Models were also effective when trained on one condition and applied to others, showing an invariance to task and stimulus type, supporting practical applications for real‐world attention tracking.

### 4.2 Broader Implications and Limitations

#### 4.2.1 Attention Modulation Across Listening Conditions

The wearable EEG system captured consistent attention‐related signatures across tasks. In SustAC, the P2 peak suggested the neural enhancement of attended signals in multi‐talker environments (Luck and Kappenman 2012). In SwitAC, attention tracking performances remained stable, indicating that wearable EEG can follow the dynamic reorientation of attention (Carta et al. 2025).

Conversational listening (ConvAC) posed greater challenges. The TRF morphology was elongated with less distinct P1 and N1 peaks compared to the single‐talker tasks. This may reflect increased cognitive load when integrating multiple auditory and visual streams. This could align with cognitive effort accounts, where resource allocation scales with task difficulty (Westbrook and Braver 2015). The conversational task required participants to track several sources of information simultaneously, possibly resulting in higher load and less transient TRF responses, pointing to a shift toward more effortful and distributed processing.

#### 4.2.2 Models Generalize Between Tasks and AV Speech Material

Our forward and backward models demonstrated robust performance across multiple variables, indicating strong generalizability. First, the methodology was largely invariant to AV speech material. Attention tracking performance for conversation material (ConvAC) was comparable to single‐talker speech (SustAC and SwitAC). This suggests that the models can capture attention‐related neural signatures across a range of speech inputs. Second, we observed invariance to the listening task. Specifically, the attention switching in SwitAC did not substantially affect the TRFs or classification performance, which remained comparable to the sustained attention task. Overall, these results suggest that a single model architecture with consistent hyperparameters can generalize across tasks and stimulus types, supporting the use of wearable EEG for real‐world attention decoding. The stability across conditions also suggests that the neural features captured by our models are robust markers of selective attention rather than being tied to a specific task or speech stimulus.

#### 4.2.3 Acoustic Envelope Versus Acoustic Onsets

Several differences between the acoustic envelope and the acoustic onset are evident from Figures 4 and 5. Across all conditions, TRFs for individual electrodes seem sharper and sparser for acoustic onsets than for acoustic envelopes, particularly for ignored speech (Figure 4D,H,L), where the responses diminished after a dominant P1 peak around 60–80 ms. This may reflect the transient nature of acoustic onsets, which capture sudden amplitude increases, versus the continuous fluctuations captured by the acoustic envelope (Rosenkranz et al. 2024).

For SustAC and SwitAC, the dominant TRF peak for the acoustic envelope seems to be P2, whereas for the acoustic onset, it was P1. This may align with the idea that the dense envelope tracks sustained stimulus dynamics, making it more sensitive to selective attention (Brodbeck et al. 2020). Interestingly, this distinction was less pronounced in frontal‐central electrodes during conversational tasks (ConvAC, Figure 4I), possibly due to the higher attentional demands reported by participants (Figure 3).

We observed a consistent latency difference of approximately 20 ms for P1, N1, and P2 between envelope and onset features across all conditions, consistent with prior TRF (Brodbeck et al. 2020). This may result from the discrete nature of onsets, which elicit earlier peaks than the corresponding envelope signals. Alternatively, onsets may act as attentional cues, directing neural processing toward specific spectrotemporal regions and facilitating early envelope tracking (Lalor et al. 2009; Haupt et al. 2025; Brodbeck et al. 2020).

Aligned with prior studies (Brodbeck et al. 2023), the acoustic onsets seem to elicit larger TRF peaks than the acoustic envelopes across all conditions. This may reflect the well‐established sensitivity of the auditory cortex to transient sound events (Daube et al. 2019). Notably, this amplitude difference was particularly pronounced for ignored speech, suggesting that responses to sudden acoustic changes remain strong even when attention is directed elsewhere, in line with earlier observations (Brodbeck et al. 2020).

#### 4.2.4 cEEGrid Performance and Potential Improvements

cEEGrid signals captured selective attention effects, but they were weaker compared to scalp EEG, limiting practical attention decoding. This aligns with previous findings, indicating that auditory decoding performance using cEEGrid currently falls behind scalp EEG (Mirkovic et al. 2016; Nogueira et al. 2019). Several factors may account for this difference. First, scalp EEG provides broader spatial coverage, particularly over central and fronto‐central regions where auditory cortical responses (e.g., N1/P2) are typically strongest (Fuglsang et al. 2017). In contrast, cEEGrid electrodes are positioned around the ear, resulting in more limited and predominantly lateralized coverage (Debener et al. 2015). Second, scalp EEG systems often include a larger number of electrodes, enabling more effective spatial filtering and noise reduction. By comparison, cEEGrid setups generally comprise fewer electrodes and reduced spatial sampling, which may in turn limit the prediction/reconstruction accuracy. Improvements to cEEGrid analysis could include optimized electrode selection, noise removal, and referencing, potentially on a subject‐specific basis given sufficient data (Holtze et al. 2022). Additionally, incorporating simultaneous scalp recordings during model training could improve the accuracy of models relying solely on cEEGrid data.

#### 4.2.5 TRFs for Conversations Versus Single Talker

As shown in Figure 9, TRFs derived from conversational material are more extended over time compared to TRFs for the single talker on the side, within the same conversation condition. In other words, the TRFs are less sparse and spread across longer time lags. This broadening may arise from overlapping acoustic features or time‐locked neural responses to the non‐attended talker included in the fitting data. Additionally, the conversational stimuli included visual information, whereas the single talker on the side did not, which may further influence TRF shape. Behavioral studies could also suggest that listening and speaking strategies adapt to the complexity of multi‐speaker environments, further affecting neural responses (Hadley et al. 2021). These differences could be considered in real‐time neural tracking applications, where perfect separation of speech streams is often not achievable.

## 5 Conclusions

Wearable EEG can reliably track selective attention in realistic listening scenarios beyond single‐talker audio‐only tasks. In this study, scalp EEG captured attention across three conditions: sustained attention to single‐talker AV stimuli, attention switching between talkers, and attending to conversational AV sources. This was evidenced by clear P2 differences in TRFs between attended and ignored speech, as well as the performance of forward and backward models. The models remained robust across conditions, showing no significant performance drop during attention switches. Notably, TRF characteristics differed between single‐talker and multi‐talker conversations, which should be considered in future research. Attention modulation was weaker for cEEGrid data, highlighting the need for further methodological improvements to reliably track auditory attention.

Classification of selective attention was above chance across all conditions using wearable EEG scalp data, with accuracies ranging from 55% to 70% for decision windows of 1.1–35 s. Backward models generalized across listening tasks and stimuli: Models trained on sustained single‐talker attention performed equally well on conversational and attention‐switching tasks. These results advance the development of neural tracking systems for realistic auditory environments.

## Author Contributions

**Johanna Wilroth:** conceptualization, investigation, methodology, software, formal analysis, data curation, visualization, validation, writing – original draft, writing – review and editing. **Oskar Keding:** conceptualization, investigation, methodology, software, formal analysis, data curation, visualization, validation, writing – original draft, writing – review and editing. **Martin A. Skoglund:** conceptualization, methodology, visualization, supervision, project administration, resources, funding acquisition, writing – review and editing. **Maria Sandsten:** conceptualization, methodology, visualization, supervision, project administration, funding acquisition, writing – review and editing. **Martin Enqvist:** conceptualization, methodology, visualization, supervision, project administration, funding acquisition, writing – review and editing. **Emina Alickovic:** conceptualization, investigation, methodology, data curation, visualization, validation, resources, funding acquisition, supervision, project administration, writing – original draft, writing – review and editing.

## Funding

This study was supported by the ELLIIT Research Programme, Sweden (C04).

## Conflicts of Interest

The authors declare the following potential conflicts of interest with respect to the research, authorship, and/or publication of this article: The commercial affiliation of authors EA and MAS does not alter our adherence to EJN's policies on sharing data and materials.

## Supporting information




**Data S1:** Supporting Information.

## Data Availability

The data underlying this study are subject to ethical and legal restrictions. Participant consent did not explicitly include permission for public data sharing, and the dataset falls under the scope of the EU General Data Protection Regulation (GDPR). For these reasons, the data are not publicly available. Data may be made available upon reasonable request, subject to institutional and ethical approval and appropriate data‐sharing agreements. The code is available at github.com/JohannaWil/audiovisual‐attention‐eeg‐trf.
