# Supplementary material for: Neural Tracking of Sustained Attention, Attention Switching, and Natural Conversation in Audiovisual Environments Using Wearable EEG
Source: Eur J Neurosci. 2026 May 8;63:e70538. doi: 10.1111/ejn.70538 (PMC13156524; doi:10.1111/ejn.70538)
Supplement: Supplementary file 1 — Data S1: Supporting Information. [file EJN-63-0-s001.pdf]

Neural Tracking of Sustained Attention, Attention Switching, and Natural Conversation in Audiovisual Environments using Wearable EEG

Johanna Wilroth, Oskar Keding, Martin A. Skoglund, Martin Enqvist, Maria Sandsten, Emina Alickovic

Information About Audiovisual Stimuli Used in Experiments

- Female single talker 1
  - **Title**: Spørg Direkte med Anne Vibeke Isaksen
  - **Author**: Spørg Direkte
  - **URL**: https://www.youtube.com/watch?v=9Ef-KMztdCs&t=4s
  - **License**: CC-BY
- Female single talker 2
  - **Title**: Spørg Direkte med Audrey Castaneda
  - **Author**: Spørg Direkte
  - **URL**: https://www.youtube.com/watch?v=QEnxtpzNbck&t=2733s
  - **License**: CC-BY
- Make single talker 1
  - Title: Spørg Direkte med Christian Have
  - Author: Spørg Direkte
  - URL: https://www.youtube.com/watch?v=Icva1hzBUMs&t=1867s
  - **License**: CC-BY
- Male single talker 2
  - **Title**: Spørg Direkte med Benjamin Koppel
  - **Author**: Spørg Direkte
  - **URL**: https://www.youtube.com/watch?v=wiXAjq7D2gI
  - **License**: CC-BY
- Conversation (Video 1)
  - **Title**: 112 For Venskaber: JEG HAR FOR MANGE VENNER
  - **Author**: HEARTBEATS DK
  - **URL**: https://www.youtube.com/watch?v=pUc-FYND65Q
  - **License**: CC-BY
- Conversation (Video 2)
  - **Title**: 112 For Venskaber: DET ER MIT BABYNAVN, OKAY?
  - **Author**: HEARTBEATS DK
  - **URL**: https://www.youtube.com/watch?v=9GECSFEwI_Q
  - **License**: CC-BY
